# Anxiety disturbs the blood plasma metabolome in acute coronary syndrome patients

**DOI:** 10.1038/s41598-021-92421-7

**Published:** 2021-06-18

**Authors:** HongYan Wei, JunYuan Gu, XueYao Jiang, Nan Deng, Jing Wu, LianHong Zou, YiMin Zhu, BoYu Tan

**Affiliations:** 1 Department of Pharmacy, The First Affiliated Hospital of Hunan Normal University (Hunan Provincial People’s Hospital), Changsha, 410005 Hunan China; 2 Department of Pharmacy, The Third Hospital of Changsha, Changsha, 410002 Hunan China; 3 Hunan Provincial Institute of Emergency Medicine, Hunan Provincial Key Laboratory of Emergency and Critical Care Metabonomics, Changsha, 410005 Hunan China

**Keywords:** Human behaviour, Acute coronary syndromes, Pharmacology, Anxiety

## Abstract

Coronary heart disease (CHD) is the result of a complex metabolic disorder caused by various environmental and genetic factors, and often has anxiety as a comorbidity. Rupture of atherosclerotic plaque in CHD patients can lead to acute coronary syndrome (ACS). Anxiety is a known independent risk factor for the adverse cardiovascular events and mortality in ACS, but it remains unclear how stress-induced anxiety behavior impacts their blood plasma metabolome and contributes to worsening of CHD. The present study aimed to determine the effect of anxiety on the plasma metabolome in ACS patients. After receiving ethical approval 26 ACS patients comorbid anxiety were recruited and matched 26 ACS patients. Blood plasma samples were collected from the patients and stored at − 80 °C until metabolome profiling. Metabolome analysis was performed by liquid chromatography mass spectrometry (LC–MS), and the data were subjected to multivariate analysis. Disturbance of 39 plasma metabolites was noted in the ACS with comorbid anxiety group compared to the ACS group. These disturbed metabolites were mainly involved in tryptophan metabolism, pyrimidine metabolism, glycerophospholipid metabolism, pentose phosphate pathway, and pentose and glucuronate interconversions. The most significantly affected pathway was tryptophan metabolism including the down-regulation of tryptophan and serotonin. Glycerophospholipids metabolism, pentose and glucuronate interconversions, and pentose phosphate pathway were also greatly affected. These results suggest that anxiety can disturb three translation of material in ACS patients. Besides the above metabolism pathways pyrimidine metabolism was significantly disturbed. Based on the present findings the plasma metabolites monitoring can be recommended and may be conducive to early biomarkers detection for personalized treatment anxiety in CHD patients in future.

## Introduction

Coronary heart disease (CHD) is the leading cause of all health loss both globally and in different world region^1^. CHD is the result of a complex metabolic disorder caused by various environmental and genetic factors^2^, and is often accompanied by psychological disease, such as anxiety^3,4^, throughout the patients’ lifelong time. Activation of the hypothalamus–pituitary–adrenal axis in CHD patients with comorbid anxiety^5^ can lead to increased blood pressure, dyslipidemia, decreased sleep quality, and increased frequency and degree of angina pectoris^6^. Increasing data from clinics has identified anxiety is an independent risk factor for the adverse cardiovascular events and mortality in patients with acute coronary syndrome (ACS)^7^. Specifically, anxiety was correlated with elevated risks for quality of life, adverse outcomes, and medical expenditure in ACS patients. Anxiety may predict 12-month non-fatal myocardial infarction and cardiac rehospitalization^8^.

The mechanism for CHD comorbidity with anxiety is complex and unclear. Previous evidences suggested that anxiety influences on neuroendocrine factors such as serotonin^9^, platelet activation, inflammation, vascular endothelial dysfunction and others^10^. Recent research revealed that changes in several metabolites, including certain amino acids, products of pyrimidine metabolism and the pentose phosphate pathway, were observed in ACS patients^2,11–13^. Metabolic disorders were also associated with anxiety^14,15^, which can aggravate coronary artery disease. Metabolites related to oxidative stress, inflammatory processes, lipid and energy metabolism, glutamine metabolism, and neurotransmission may have the potential to serve as biomarkers for anxiety disorders^16^. Lipid oxidative stress, inflammation, and abnormal energy metabolism in comorbid patients^17,18^ accelerate atherosclerosis^19^. Progression of coronary artery structural vulnerable plaque to functional vulnerable plaque, secondary plaque rupture, and thrombosis lead to ACS and myocardial infarction^20^.

Metabonomics is an important part of system biology. Its research methods are systematic, dynamic, and sensitive, and provide effective research method for exploring the pathogenesis of diseases. Among the analytical platforms of metabolomics, liquid chromatography mass spectrometry (LC–MS) is a potential tool for identifying biomarkers that can be used better risk classification and for understanding the pathophysiological processes of CHD and anxiety^21,22^. Meanwhile, LC–MS has increased the number of lipid classes that can be analyzed, separated, and identified trace components of complex^23^. However, there are few studies on metabonomics of ACS comorbid anxiety disorder, and only one study on targeted lipid metabonomics in CHD patients with comorbid depression^24^. Thus, the present study will reveal the whole functional state of the organism and the response rule to external stimulation by LC–MS detection of the changes in endogenous substances in ACS patients to search for biomarkers for ACS with comorbid anxiety.

## Materials and methods

### Study design and participants

To ensure homogeneity for all clinical features except the Hamilton Anxiety Scale (HAMA) score, the ACS group was selected. Sample size estimation was made using the MetaboAnalyst tool, based on this pilot data set. We calculated that approximately 20 samples per group would afford the study robustness of approximately 0.8 (enter 100 as the maximum sample size per group; leave the FDR cutoff as 0.1, Supplementary Figure S1). The study was carried out at the First Affiliated Hospital of Hunan Normal University, which admitted about 438 CHD inpatients in cardiovascular department in 2019. Based on our predefined entry criteria as following, we recruited 26 ACS comorbidity with anxiety inpatients, and 26 propensity matched non-anxiety ACS inpatients. The process of patient selection was shown in Supplementary Figure S2.

A questionnaire survey for personal assessment of anxiety was conducted for these patients. Complete questionnaires finished within the first day after admission were recognized as eligible and included in the following analysis. Incomplete questionnaires and participants with a history of psychological or cognitive disorder were excluded. The baseline data of the inpatients were extracted from the electronic medical record of Hunan Provincial People's Hospital according to the inclusion criteria and exclusion criteria as described below. The study's protocol was established according to the ethical guidelines of the Helsinki Declaration and was approved by the Human Ethics Committee of Hunan Provincial People's Hospital/First Affiliated Hospital of Hunan Normal University (No. 2018-20). The inclusion criteria for patients with ACS were (1) diagnosis of CHD in accordance with the WHO diagnostic standard in 1999, and confirmed as ACS, including acute myocardial infarction, by electrocardiogram, color doppler echocardiography, and coronary angiography; (2) no prior administration of anti-anxiety and anti-depression drugs taking before; (3) normal reading and cognitive ability, and cooperation to fill out the HAMA assessment; (4) HAMA score ≤ 14; (5) age ranges from ≥ 18 to ≤ 80 years; and(6) resting blood pressure value ≤ 180/120 mmHg.

The exclusion criteria were: (1) cardiac function grading of New York Heart Association (NYHA) III grade or higher; (2) comorbidity with severe arrhythmia or severe cardiac dilatation; (3) resting blood pressure > 180/120 mmHg; (4) comorbidity with diabetes or blood sugar not showing improvement after treatment; (5) complication with chronic infectious diseases or serious liver, brain, kidney or lung related diseases; (6) history of depression or anxiety, and use of psychotropic drugs, alternative drugs, or psychotherapy within the first 4 weeks or inclusion in electric shock treatment within 8 weeks previously, and substance abuse or dependence within the first 3 months; (7) severe anxiety or depression, cognitive impairment, nervous system disease, or other mental illness; (8) traumatic heart surgery within 3 months; (9) presence of tumors; and (10) abnormal thyroid function.

In addition to the above diagnostic criteria, patients with ACS comorbid anxiety met the diagnostic criteria of Chinese Classification and Diagnostic Criteria of Mental Disorders (CCMD-3), and the international HAMA score ≥ 14^25^.

The data collected for the patients included age, sex, diagnosis, chronic diseases like hypertension (yes/no) and hyperlipidemia (yes/no), percutaneous coronary intervention (PCI) (yes/no), platelet count, left ventricular ejection fraction (LVEF), fasting blood glucose (FBS), triglyceride (TG), cholesterol (TC), low-density lipoprotein cholesterol (LDL-C), and hepatic and renal functions. The above data were extracted as baseline demographic characteristics, and HAMA score was supplied to evaluate the degree of anxiety in ACS patients after informed consents were obtained from all patients.

### Questionnaire measurement of anxiety

The HAMA scale has been widely used to assess the appearance of anxiety^26,27^. The HAMA scale contains 14 questions, and each question has 5 items. Responses are scored as 0 (never), 1 (mild), 2 (moderate), 3 (severe), or 4 (extremely serious). The total score of the HAMA scale is operationally categorized as follows: no anxiety (score 0–6), possible anxiety (score 7–13), and definite anxiety (score ≥ 14). Various previous studies have demonstrated that these questionnaires can assess the psychological condition with satisfactory reliability and validity.

### Collection, treatment, and analysis of blood samples

#### Biochemical assay

Plasma samples were collected prospectively from the propensity score matching cohort including 21 ACS patients with comorbid anxiety and 26 ACS patients. Blood was collected from veins, kept in heparinized tubes, and centrifuged at 3000×*g* at 4 °C for 5 min to obtain plasma. Plasma neuropeptide Y(NPY) levels were measured by enzyme linked immunosorbent assay (CUSABIO, USA) in accordance with the manufacturer’s instructions.

#### Chemicals

LC–MS grade acetonitrile and HPLC grade methanol were purchased from Merck (Darmstadt, Germany). Methanoic acid was purchased from CNW Company (Germany). All other chemicals were of analytical grade and were purchased from Sigma (St. Louis, MO, USA). Watson’s distilled water was used.

### Metabolome analysis using ultra-performance liquid chromatography coupled with quadrupole time-of-flight tandem mass spectrometry (UPLC-QTOF-MS)

Blood samples were collected as described above, and the plasma supernatants were stored at − 80 °C until the metabolome profiling was performed. All samples were thawed for 15 min and vortexed for 5 s prior to analysis. A 200 µL aliquot of each plasma sample was mixed with 600 µL of methanol, vortexed for 40 s, and left undisturbed for 20 min. After centrifugation at 12,000×*g* for 15 min at 4 °C, 600 μL of supernatant was dried under a vacuum at room temperature. The obtained residue was dissolved in 200 μL of 50% acetonitrile, vortexed for 40 s, and centrifuged at low temperature (4 °C) and high speed (12,000 rpm) for 10 min. Finally, 100 µL of supernatant was subjected for the UPLC-QTOF-MS assay.

UPLC-QTOF-MS analyzer (Impact II; Bruker) was used to analyze the samples. The chromatographic separation conditions and quality control (QC) procedures were based on the experimental methods of Ren et al.^28^. Briefly, each sample (10 µL) was injected into an Acclaim TM 120 C_18_ column (Thermo Fisher, USA, 100 × 2.1 mm, 2.2 µm) at 4 °C, and the following mobile phases were used at a flow rate of 0.2 mL/min at 30 °C: H_2_O with 0.1% methane acid (A) and acetonitrile with 0.1% methane acid (B). Mass spectrometry analysis was performed in the V flight tube detection mode with nitrogen as the atomization cone gas in both the positive and negative ion modes. The source temperature was set at 200 °C, the extraction cone was set at 4 V, and a cone gas flow of 8.0 L/min was used in both modes. The capillary voltage was set at 4.0 and 3.5 kV, the sampling cone was set at 35 and 50 kV, the desolvation temperature was set at 350 °C and 300 °C, and the desolvation gas flow was set at 600 and 700 L/h, respectively. Mass spectrometric data were collected in the centroid mode from 20 to 1000 m/z.

The peak height for the internal standard was continuously monitored during the analysis to ensure signal stability. The QC procedures were employed to validate the methods and ensure stability. For this, 50 µL aliquots of QC samples were prepared by pooling identical volumes to the individual plasma samples. To ensure that the system was suitable for use, six pooled QC samples were run prior to the analysis in each ion mode. Six ions (min_m/z) were selected to evaluate the relative standard deviation (RSD) of the retention time, m/z, and peak area.

**Figure 1:**
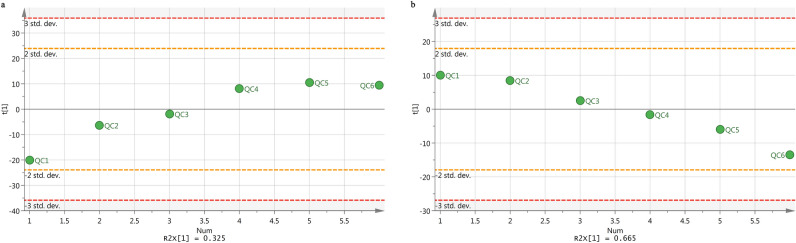
The first main dispersion point diagram of QC sample using LC–MS method (**a** positive mode; **b** negative mode).

The scatter plot of the first principal component is shown in Fig. 1. The results confirmed that all QC samples were distributed within the scope of 2SD, indicating that the consistency of the experimental operation and the stability of the instrument system were within a controllable range.

### Identification of metabolites

The UPLC-QTOF-MS data were imported into the Metaboscape version 3.0 analysis software (Bruker Company), and positive and negative Brooke matrix tables were established to detect and align the peaks of all samples. The parameters were set as follows: chromatographic peaks with retention time of 0–30 min were intercepted; the peak intensity threshold was 1000; the minimum peak length was 5 spectra; the peaks were screened according to the 80% rule; and the mass spectrum data were corrected with sodium formate. After being identified and aligned, we normalized the strength of each ion with the total strength of all ions in each chromatogram. We used the standard database, HMDB database, and online search database of Bruker Company to identify the secondary mass spectra and obtain the corresponding compounds. Finally, the three-dimensional matrix information included the retention time (RT), mass/charge ratio (m/z), ionic strength data (variables), and compound name. In addition, Kyoto encyclopedia of genes and genomes (KEGG; http://www.genome.jp/kegg/) biochemical database were used to interpret possible pathways involving the identified metabolites.

### Statistical analysis

Continuous variables were expressed as mean ± standard error of the mean, and compared by Student’s *t*-test before the Levene test to ensure equality of variances, otherwise using the Wilcoxon rank-sum test. Categorical variables were expressed as number (percentage), and compared by the chi-square test. All statistical analyses were performed with SPSS 22.0 software. Significant differences were accepted for values of *p < 0.05.

Mass Profile software was used for peak extraction, RT alignment, peak alignment, and deconvolution analysis. Finally, data were imported into SIMCA-P software (v14.0; Umetric, Umea, Sweden) for the PCA and OPLS-DA. VIP > 1 and *p < 0.05 were considered to indicate statistical significance.

### Ethics statement

The experiment protocol was approved by the Medical ethics committee of Hunan Provincial People's Hospital/First Affiliated Hospital of Hunan Normal University (Experiment License: 2018-20).

## Results

### Baseline characteristics of the enrolled patients

A total of 438 CHD inpatients in our cardiology department were screened in 2019, and 226 ACS patients met the enrolled criteria, comprising 26 ACS patients with comorbid anxiety and 200 ACS patients. One-to-one propensity score matching (PSM) created 26 pairs. Five outliers were subsequently removed from the metabonomics analysis in the ACS with comorbid anxiety group, and thus 21 patients were finally included in this group.

The study included CHD patients who were hospitalized for the first time because of ACS. The enrolled patients had a mean age of > 60 years and complications of hypertension and/or hyperlipidemia. None of the patients had serious liver or kidney disfunction, diabetes, thyroid disease, and other metabolic abnormalities. The mean values of TG and LDL-C had significant differences (*p* ≤ 0.05) between the two groups before PSM, while there were no significant differences (*p* ≥ 0.05) in the baseline data after PSM. The demographic characteristics for all participants before and after PSM were presented in Table 1.

**Table 1:** Demographic information of the enrolled participants about angxiety comorbidity with ACS and ACS group before and after PSM.

Parameters	Pre-matched groups		*P*	Matched groups		*P*
	Angxiety with comorbid ACS group (n = 26)	ACS group (n = 200)		Angxiety with comorbid ACS group (n = 26)	ACS group (n = 26)	
Age (year), mean (SD)	62.92 (10.62)	65.41 (9.75)	0.7	62.92 (10.62)	62.62 (8.14)	0.38
Male, n (%)	12 (46.15)	124 (62.00)	0.48	12 (46.15)	12 (48.00)	0.57
Hypertension, n (%)	13 (0.50)	137 (68.50)	0.45	13 (0.50)	18 (72.00)	0.75
Hyperlipidemia, n (%)	10 (38.46)	44 (22.00)	0.02*	10 (38.46)	8 (32.00)	0.76
PCI, n (%)	8 (30.77)	110 (55.00)	0.11	8 (30.77)	8 (30.77)	0.78
LVEF, mean (SD)	56.10 (15.20)	57.29 (13.42)	0.89	56.10 (15.20)	53.25 (14.33)	0.43
PLT (×10^9^/L), mean (SD)	188.00 (59.08)	200.70 (59.95)	0.12	183.89 (61.10)	215.56 (73.45)	0.33
FBS (mmol/L), mean (SD)	5.45 (1.85)	5.76 (2.45)	0.2	5.45 (1.89)	4.91 (3.01)	0.06
TC (mmol/L), mean (SD)	3.90 (1.41)	3.95 (1.07)	0.67	3.47 (1.84)	4.13 (1.47)	0.58
TG (mmol/L), mean (SD)	2.28 (3.25)	1.70 (1.32)	0.01*	2.01 (3.21)	1.56 (1.14)	0.17
LDL-C (mmol/L), mean (SD)	2.13 (0.73)	2.25 (0.89)	0.01*	1.87 (0.99)	2.84 (1.18)	0.38
Bun (mmol/L), mean (SD)	6.15 (3.13)	6.84 (7.77)	0.49	6.36 (3.21)	5.18 (1.62)	0.18
Cr (µmol/L), mean (SD)	89.00 (45.71)	92.50 (92.12)	0.46	88.24 (44.51)	71.64 (24.11)	0.32
ALT (u/L), mean (SD)	23.13 (9.33)	26.61 (25.23)	0.12	21.80 (9.38)	29.84 (31.18)	0.22
TP (g/L), mean (SD)	60.54 (4.64)	61.29 (6.21)	0.08	61.13 (5.37)	61.97 (4.65)	0.56
ALB (g/L), mean (SD)	38.62 (2.71)	38.91 (4.20)	0.06	38.318 (2.43)	38.53 (3.12)	0.62

SD, standard deviation; PSM, propensity score matching; *p < 0.05.

**Figure 2:**
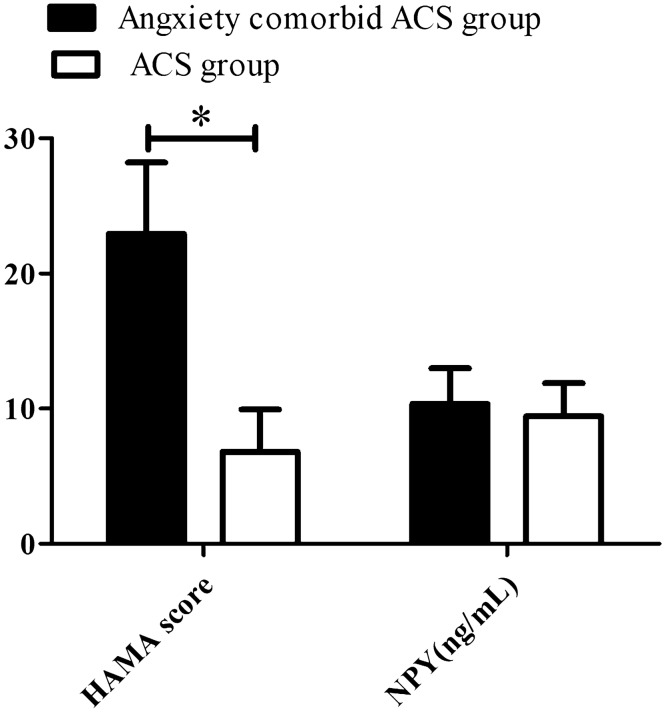
Effect of anxiety on HAMA score and NPY in ACS patients. Data are presented as mean ± SD (*p < 0.05).

As shown in Fig. 2, the HAMA score in the ACS with comorbid anxiety group was significantly higher than that in the ACS group, but there was no significant difference in serum NPY between the two groups.

### Metabonomics results for UPLC-QTOF-MS

#### Base peak chromatogram (BPC) under positive and negative ion conditions

**Figure 3:**
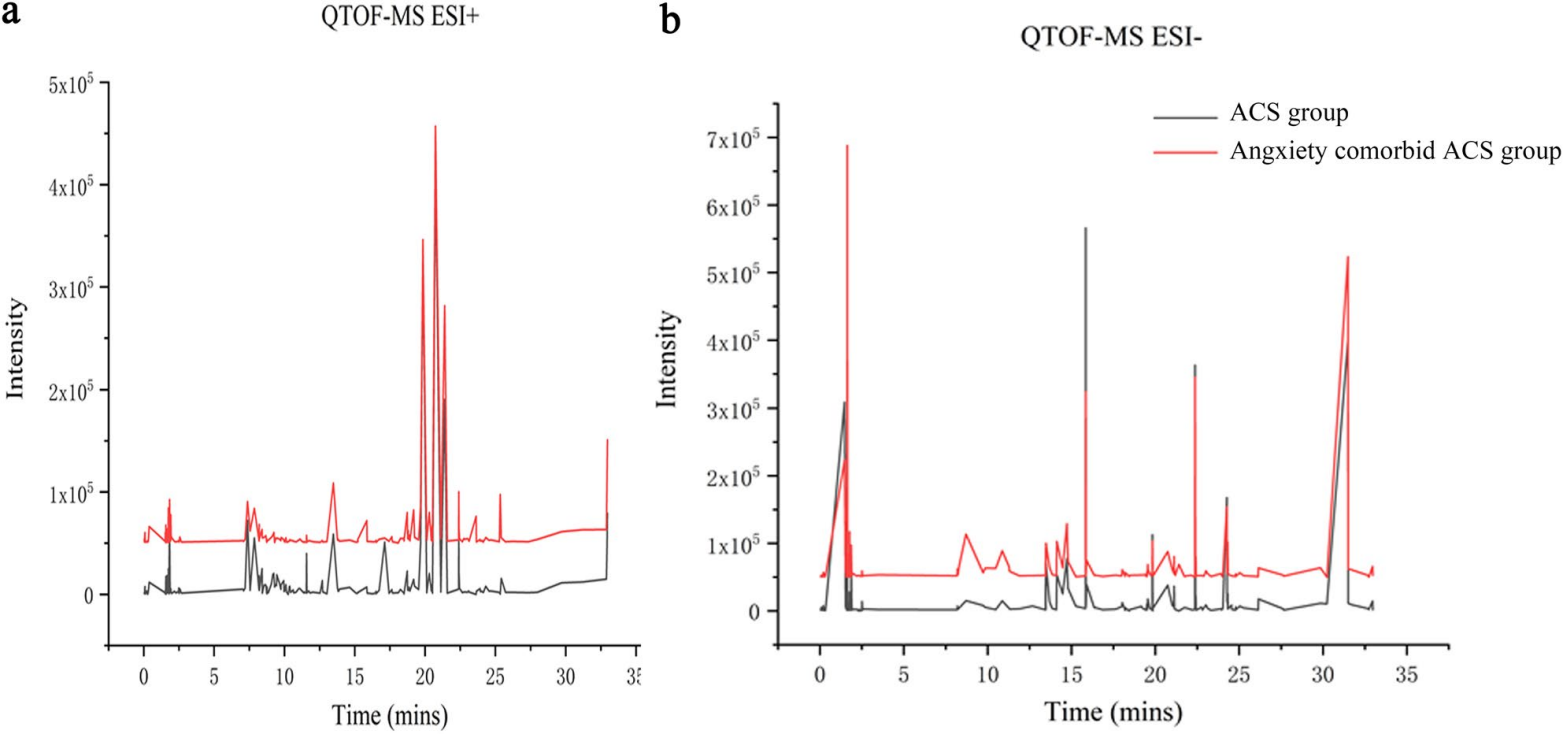
Representative LC–MS analysis of total ion chromatograms for plasma. Plasma samples in positive (**a**) and negative mode (**b**).

The original data were drawn with Origin 2017, and the chromatogram as shown in Fig. 3 was obtained. As can be roughly seen, the characteristics of the metabolites under the two modes were basically similar, but their responsivities were different. A total of 415 positive ions and 420 negative ions were identified using the HMDB database and PubChem database.

#### Principal component analysis (PCA)

**Figure 4:**
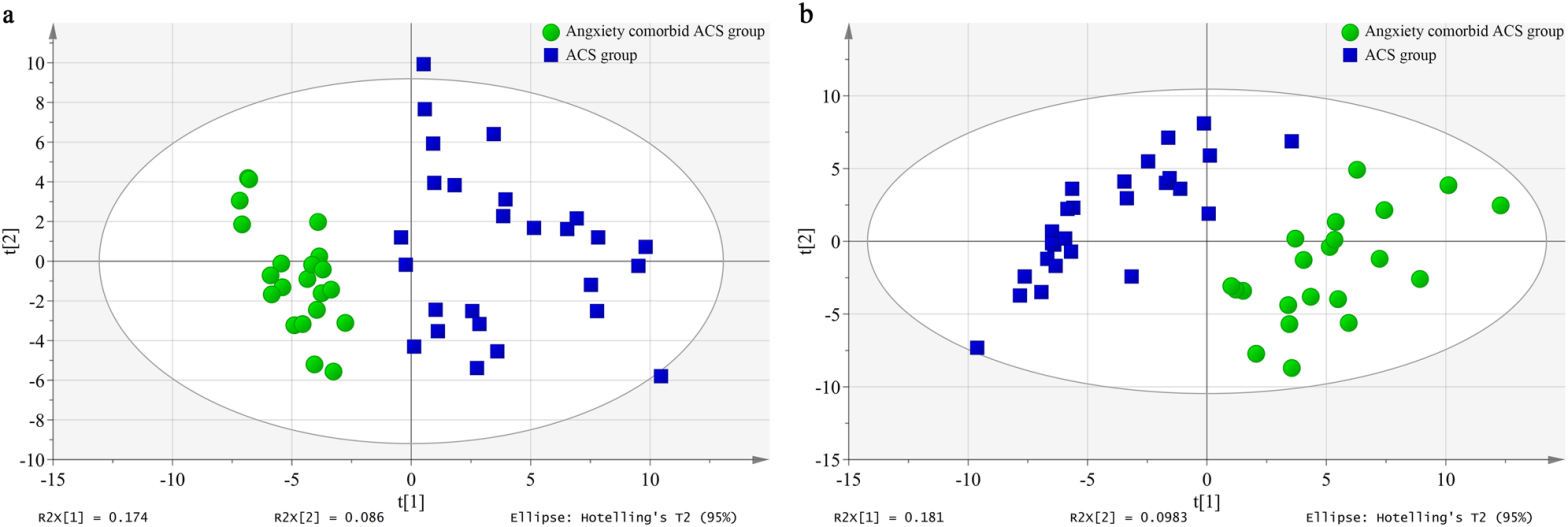
PCA score plots derived from LC–MS analysis of plasma from anxiety comorbidity ACS and ACS group in different ion modes. Plasma in positive ion mode (**a**), in negative ion mode (**b**).

An unsupervised PCA was performed to provide an overview of the LC–MS data. The PCA score plots are shown in Fig. 4. Significant differences were observed between two groups for the plasma samples in both ion modes.

### Orthogonal partial least squares discriminant analysis (OPLS-DA)

**Figure 5:**
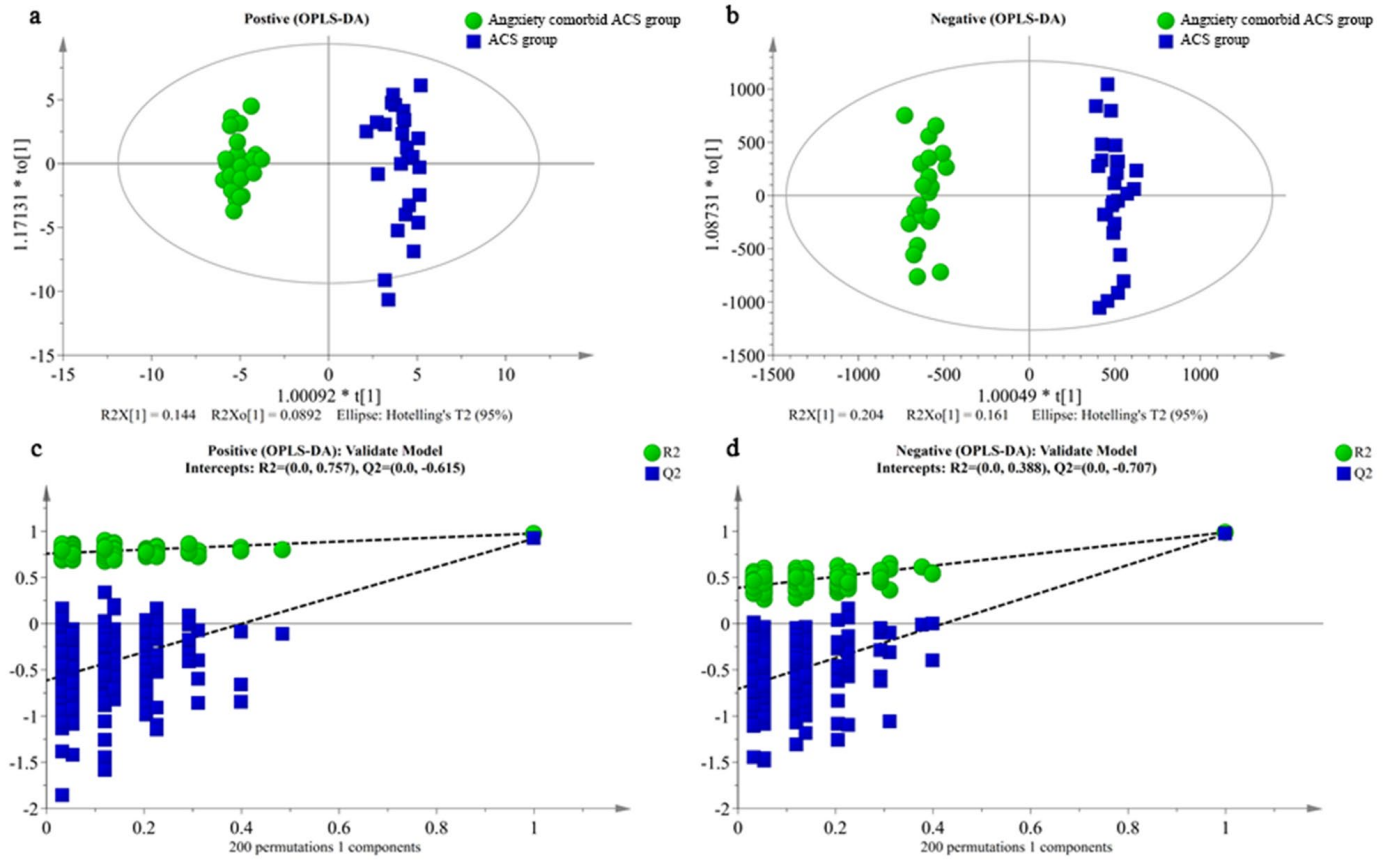
The OPLS-DA score plots and sorting validation figures derived from LC–MS analysis of plasma from anxiety comorbidity ACS and ACS group in positive ion mode (**a**,**c**), and in negative ion mode (**b**,**d**).

An OPLS-DA model was constructed. The key model parameters are summarized in Fig. 5a,b. The results for the groups were visualized as score plots to show any group clusters. S-plots were used to identify variables that contributed to the classification. Good separation between the groups was observed. Measures for the quality of the resulting discrimination, including the R2X and Q2 values, are shown in Fig. 5c,d. The R2 and Q2 values were > 0.5, indicating good fitness and prediction.

### Anxiety disturbs the metabolome in ACS patients

**Figure 6:**
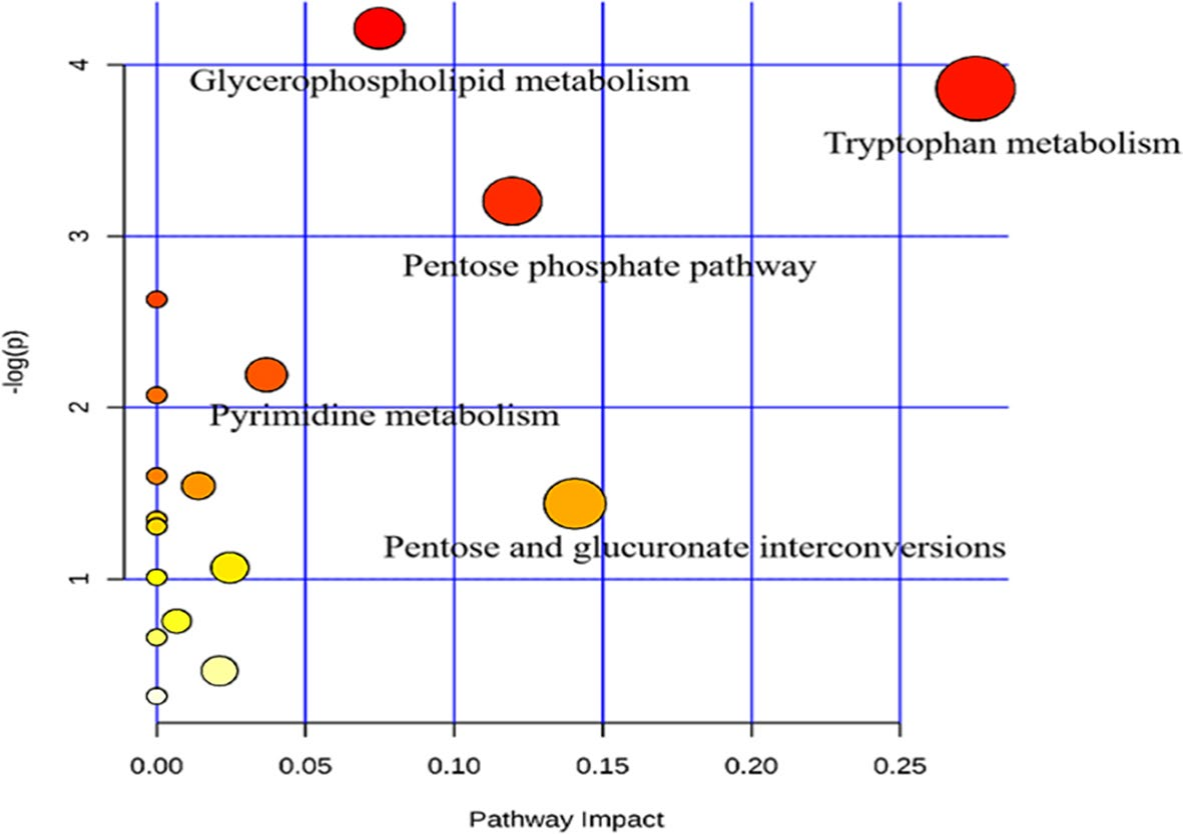
The differential metabolites of plasma involved in main pathways between anxiety comorbidity ACS and ACS group.

Differentially-expressed metabolites were identified based on the variable importance in projection (VIP) with a threshold of 1.0 for the OPLS-DA model and *p* < 0.05 in the *t*-test. Metabolites were identified based on mass assignment and identified ion (m/z) and retention time (RT), and were then compared with authentic standards or database resources, such as KEGG (http://www.genome.jp/kegg/) and METLIN (http://metlin.scripps.edu). Among hundreds of metabolites, 39 molecules in the plasma samples were significantly correlated with anxiety in ACS patients (Table 2). Specifically, in the plasma samples of the ACS with comorbid anxiety group, the levels of 3-alpha-androstanediol glucuronide, serotonin, 3-hydroxycapric acid, 25-hydroxyvitamin D2, androsterone sulfate, 5a-tetrahydrocorticosterone, 5-androstenetriol, beta-alanine, 4-hydroxycyclohexylcarboxylic acid, tryptophan, gamma-glutamylthreonine, 5-hydroxydantrolene, 3-carbamoyl-2-phenylpropionaldehyde, 4-hydroxynonenal, alpha-carboxyethyl hydroxychroman, aminoethoxyacetic acid, isobutyryl-l-carnitine, phosphoribosyl pyrophosphate, and phosphorylcholine were decreased, while the levels of lysophosphatidylcholine, oleoylcarnitine, 19-hydroxyandrost-4-ene-3,17-dione, tetrahydrocortisone, 17-hydroxypregnenolone sulfate, 11-oxo-androsterone glucuronide, 7-methylguanine, deoxycholic acid 3-glucuronide, glycerophosphocholine, hippuric acid, l-tryptophan, thromboxane B2, and trans-aconitic acid were increased. These data suggest that changes mainly occurred in the following metabolic pathways in the ACS with comorbid anxiety group (Fig. 6): tryptophan metabolism, glycerophospholipid metabolism, pentose phosphate metabolism, pyrimidine metabolism, and pentose and glucuronate interconversion.

**Table 2:** Metabolites and pathways in plasma samples that differed between the anxiety comorbidity with ACS and ACS group.

Metabolites	Ionization mode	VIP	RT (min)	m/z	Fold change (A/C)	*p*-value	Metabolic pathway	KEGG ID
3-Alpha-androstanediol glucuronide	ESI−	1.06	24	467.27	− 2.41	0.00		
Serotonin	ESI−	2.14	15.85	175.28	− 2.19	0.00	Tryptophan metabolism	C00780
3-Hydroxycapric acid	ESI−	1.37	22.75	187.15	− 2.02	0.00		
25-Hydroxyvitamin D2	ESI−	1.87	22.39	411.33	− 1.82	0.00		
Androsterone sulfate	ESI−	1.51	22.16	367.18	− 1.80	0.04		
5a-Tetrahydrocorticosterone	ESI−	1.42	22.36	349.22	− 1.54	0.00		
5-Androstenetriol	ESI−	3.16	24.27	305.20	− 1.31	0.00		
Beta-alanine	ESI−	1.22	1.60	88.04	− 1.13	0.01	Pyrimidine metabolism	C00099
4-Hydroxycyclohexylcarboxylic acid	ESI−	1.73	1.61	143.08	− 1.12	0.03		C04404
Tryptophan	ESI−	1.94	1.61	101.05	− 1.09	0.03	Tryptophan metabolism	C00078
Gamma-glutamylthreonine	ESI−	4.33	1.61	123.04	− 1.08	0.01		
5-Hydroxydantrolene	ESI−	3.45	1.63	164.01	− 1.049	0.00		
LysoPC(20:5(5Z,8Z,11Z,14Z,17Z))^a^	ESI−	1.74	20.74	540.33	0.82	0.00	Glycerophospholipid metabolism	C04230
Oleoylcarnitine	ESI−	5.00	31.49	424.33	0.75	0.01		
19-Hydroxyandrost-4-ene-3,17-dione	ESI−	1.08	22.43	301.1661	0.73	0.01	Steroid hormone biosynthesis	C05290
Tetrahydrocortisone	ESI−	1.63	24.32	363.20	0.69	0.01		
^a^LysoPC(16:0)	ESI−	1.18	21.01	478.29	0.64	0.00	Glycerophospholipid metabolism	C04230
^a^LysoPC(15:0)	ESI−	1.16	23.02	480.3096	0.64	0.00	Glycerophospholipid metabolism	C04230
^a^LysoPC(22:6(4Z,7Z,10Z,13Z,16Z,19Z))	ESI−	1.61	21.4	566.35	0.60	0.00	Glycerophospholipid metabolism	C04230
17-Hydroxypregnenolone sulfate	ESI−	1.38	23.92	411.21	0.45	0.00		
11-Oxo-androsterone glucuronide	ESI+	3.79	9.95	481.26	0.10	0.02		
3-Carbamoyl-2-phenylpropionaldehyde	ESI+	1.14	0.27	194.12	− 3.40	0.00		
4-Hydroxynonenal	ESI+	2.99	9.25	139.08	− 1.88	0.02		
7-Methylguanine	ESI+	1.45	1.54	83.54	0.48	0.03		C02242
Alpha-carboxyethyl hydroxychroman	ESI+	2.93	22.4	301.14	− 1.43	0.00		
Aminoethoxyacetic acid	ESI+	1.53	6.89	120.08	− 2.06	0.00		
Beta-alanine	ESI+	1.15	25.74	90.09	− 1.36	0.03	Pyrimidine metabolism	C00099
Deoxycholic acid 3-glucuronide	ESI+	3.28	10.34	569.31	0.08	0.03	Pentose and glucuronate interconversions	C03033
Glycerophosphocholine	ESI+	2.12	1.76	280.10	0.735	0.01	Glycerophospholipid metabolism	C00670
Hippuric acid	ESI+	1.65	1.52	90.52	0.70	0.01	Phenylalanine metabolism	C01586
Isobutyryl-l-carnitine	ESI+	1.10	7.2	232.15	− 2.26	0.04		
l-Tryptophan	ESI+	1.63	12.56	205.13	0.66	0.00	Glycine, serine and threonine metabolism	C00078
LysoPC(18:0)^a^	ESI+	2.41	23.63	524.37	0.39	0.00	Glycerophospholipid metabolism	C04230
LysoPC(18:1(11Z))^a^	ESI+	2.92	21.39	544.34	0.81	0.01	Glycerophospholipid metabolism	C04230
LysoPC(22:5(4Z,7Z,10Z,13Z,16Z))^a^	ESI+	1.14	20.3	592.34	0.69	0.01	Glycerophospholipid metabolism	C04230
Phosphoribosyl pyrophosphate	ESI+	1.06	1.62	390.99	− 1.74	0.00	Pentose phosphate pathway	C00119
Phosphorylcholine	ESI+	2.23	8.19	207.11	− 1.49	0.00	Glycerophospholipid metabolism	C00588
Thromboxane B2	ESI+	3.71	9.46	393.21	0.12	0.03		C05963
Trans-aconitic acid	ESI+	1.85	0.08	88.02	0.23	0.03	C5-Branched dibasic acid metabolism	C02341

^a^*LysoPC* lysophosphatidylcholine.

## Discussion

Patients with CHD often have comorbid anxiety. Chronic anxiety stress can cause unstable atherosclerotic plaque, myocardial ischemia, and even acute myocardial infarction. However, the international HAMA score lack an objective diagnostic basis for clinical diagnosis of CHD. In the present study, LC–MS was used to detect and analyze serum metabolites in ACS patients with comorbid anxiety to obtain new objective evidence for the diagnosis of this comorbidity.

The baseline characteristics of the two groups evaluated in the study were essentially the same after PSM. In consideration of the physiological roles of Neuropeptide Y(NPY) for blood pressure, atherogenic processes, and anxiety^29,30^, the levels of NPY were examined. The results showed that the NPY level was slightly increased in the ACS with comorbid anxiety group compared with the ACS group, but the difference was not significant. This finding is not consistent with other research^31^, which may be related to the lack of specificity in the biological functions of NPY^32^.

By LC–MS metabonomics and OPLS-DA analyses, the 39 metabolites identified as being disturbed in plasma samples from the ACS with comorbid anxiety group compared with the ACS group (Table 2) were involved in multiple pathways (Fig. 6). The most significantly affected pathway was tryptophan metabolism including down-regulation of tryptophan and serotonin, which are closely related. The starting point of this pathway is tryptophan. As the only precursor of serotonin, increased tryptophan consumption in the central nervous system leads to corresponding enhancement of the serotonin level and activity in the brain^33^. However, an abnormal decrease in the plasma tryptophan content, such as the occurrence of acute tryptophan depletion (ATD), can lead to anxiety behavior. ATD can cause the increase of sympathetic nerve activity, and the decrease of parasympathetic nervous system activity. These effects are positively correlated with the anxiety score of patients^34^. Serotonin is a monoamine that acts as a neurotransmitter and neuromodulator, affecting cognitive and emotional abilities^35^. Based on examinations of gene expression and transporter activity, it has been proven that decreases in the serotonin system are related to a variety of mental diseases, with serotonin identified as a key component of anxiety^36^.

Inflammation increases platelet activation, which has important roles in thrombosis and myocardial ischemia^37^. 5-Hydroxytryptamine was shown to increase platelet aggregation, and anxiety is associated with 5-hydroxytryptamine system abnormalities^38^. Serotonin binds 5-hydroxytryptamine-2 (5ht-2) receptor on platelets and precipitates factors that enhance platelet aggregation. In healthy blood vessels, nitrous oxide prevents thrombosis by releasing into the endothelium and subsequent induction of vasodilation. However, when atherosclerotic diseases damage endothelial cells, blood vessels are unable expand properly, and exposure to 5-hydroxytryptamine can resulted in vasoconstriction. This may be the underlying mechanism for the association between increased blood serotonin levels and cardiac events in CHD patients^14^. In the present study thromboxane B2 metabolism was increased in the ACS with comorbid anxiety group. This suggests that there may be abnormalities in platelet serotonin (5-HT) receptors that enhance the response of platelets to 5-HT, and the release of 5-HT promotes platelet aggregation. In addition, an imbalance of thromboxane A2 prostacyclin can cause vasoconstriction, which can further promotes the occurrence of heart thrombosis.

In addition to tryptophan metabolism, glycerophospholipid metabolism was also found to be greatly affected in the present study. Specifically, phosphatidylcholine was down-regulated, while glycerophosphate choline was up-regulated in the ACS with comorbid anxiety group. Our study identified seven compounds belonging to the glycerophospholipid metabolism pathway. These compounds, namely (16:0), lysoPC (15:0), lysoPC (20:5 (5Z, 8Z, 11Z, 14Z, 17Z), lysoPC (22:6(4Z,7Z,10Z,13Z,16Z,19Z)), lysoPC (18:0), lysoPC (18:1(11Z)), and lysoPC (22:5 (4Z, 7Z, 10Z, 13Z, 16Z)) were all up-regulated. In studies on mice, dogs, and other animals, phospholipid metabolism disorders were common. Puurunen and other non-target LC-QTMF-MS were used to analyze the whole blood samples of frightened and anxious dogs. Under the same diet control, six glycerophospholipid metabolism disorders were decreased in the anxiety group^39,40^. Robert et al. analyzed the plasma phospholipid spectra of 31 mice models with LC, hydrophilic interaction liquid chromatography and high-resolution mass spectrometry, and found that the most obvious up-regulation in phosphatidylinositol (PE) and lysophosphatidylethanolamine (LPE)^14^. Although these phospholipids differ from the phospholipid compounds identified in the present study, they all provide theoretical support for the hypothesis that membrane lipids play a key role in anxiety-related diseases in animals. As components of the cell membrane, lipids determine the location and function of various receptors that may represent useful biomarkers in the analysis of emotions. Meanwhile phosphatidylethanolamine ceramide was identified as a biomarker for CHD diagnosis in a metabonomics study^41^. Therefore, further clinical studies are warranted to determine phospholipid compounds that may be more accurate and helpful in the diagnosis of patients with CHD and comorbid anxiety. Lipid metabonomics may also provide a valuable direction for anxiety research in the future. Identified metabonomics biomarkers will help toward early diagnosis and treatment of ACS patients with comorbid anxiety, and reduce the rehospitalization rate and mortality rate of these patients.

Hyperlipidemia is closely related to stress and anxiety^42^. Sympathetic activation in generalized anxiety disorder increases lipoprotein lipase activity through the release of adrenaline and corticosteroids^14^. The resulting hyperactivity in lipoprotein lipase results in an increase in free fatty acids that can be converted into cholesterol and triglycerides. However the present results indicated that 19-hydroxy-androsterol-4-ene-3, 17-dione and tetrahydrocortisone were down-regulated, suggesting that steroid hormone biosynthesis and cortisone metabolism were decreased in CHD patients with comorbid anxiety. Low baseline cortisol was reported in people at risk for developing post-traumatic stress disorder and low corticosterone response to stressful and anxiogenic stimuli^43^. The present results also showed that anxiety caused by long-term stress can reduce a corticosterone response in patients with CHD.

In the present study phosphoribosyl pyrophosphate and β-alanine were down- regulated, while 7-methylguanine and hippuric acid were up-regulated. Phosphoribosyl pyrophosphate is an important metabolic intermediate in the pentose phosphate pathway, which is a vital pathway for oxidative decomposition of glucose. Its function is not to produce ATP, but to produce special substances with important physiological functions, such as NADPH and 5-ribose phosphate. The pentose phosphate pathway has effects on the metabolism in ischemic heart disease^44^. It is also involved in the de novo and remedial synthesis of purine, pyrimidine nucleotides, and certain amino acids such as tryptophan. These substances all promote progression of atherosclerosis.

In addition to tryptophan metabolism, phospholipid metabolism, pentose phosphate metabolism, and steroid hormone biosynthesis disturbance-related compounds, 25-hydroxyvitamin D2 was down-regulated by 1.82 times. A large decline of vitamin D2 was reported to be correlated with anxiety^45^. Vitamin D supplementation in rats helped to regulate and protect the dopamine system, and played a role in anti-anxiety^46^. Therefore, 25-hydroxyvitamin D2 supplementation may help to reduce anxiety. Of course, the present study was an exploratory study based on metabonomics research in a small population of patients with ACS and comorbid anxiety, and thus the differential metabolites between the two groups require further verification in vivo and in vitro.

## Conclusions

There were notable metabolic differences in ACS patients with comorbid anxiety compared with ACS patients in the present study. The altered small-molecule metabolites were mainly attributed to tryptophan metabolism, steroid hormone biosynthesis, pyrimidine metabolism, phenylalanine metabolism, glycerophospholipid metabolism, and pentose phosphate metabolism pathways. Identification of these biomarkers and their pathways will help to unravel the molecular mechanism of comorbid anxiety in CHD patients, and thereby facilitate early diagnosis, accurate disease classification, and personalized treatment of these patients.

## Supplementary Information

Supplementary Information.
