# Supplementary material for: Anxiety disturbs the blood plasma metabolome in acute coronary syndrome patients
Source: Sci Rep. 2021 Jun 18;11:12897. doi: 10.1038/s41598-021-92421-7 (PMC8213718; doi:10.1038/s41598-021-92421-7)
Supplement: Supplementary file 1 — Supplementary Information. [file 41598_2021_92421_MOESM1_ESM.pdf]

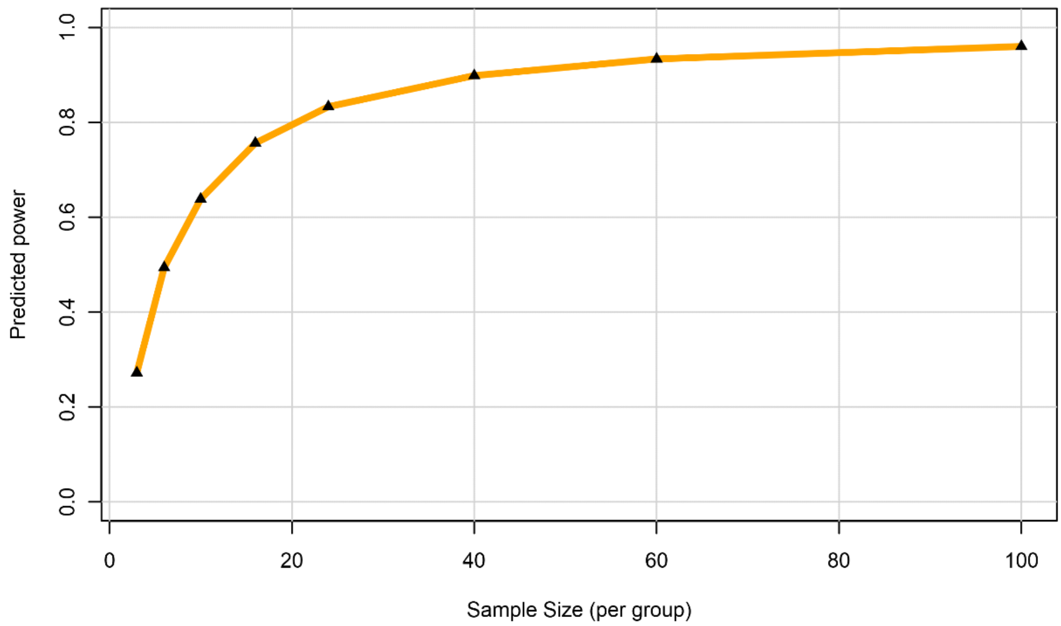

Supplementary Figure 1 Sample size estimation.

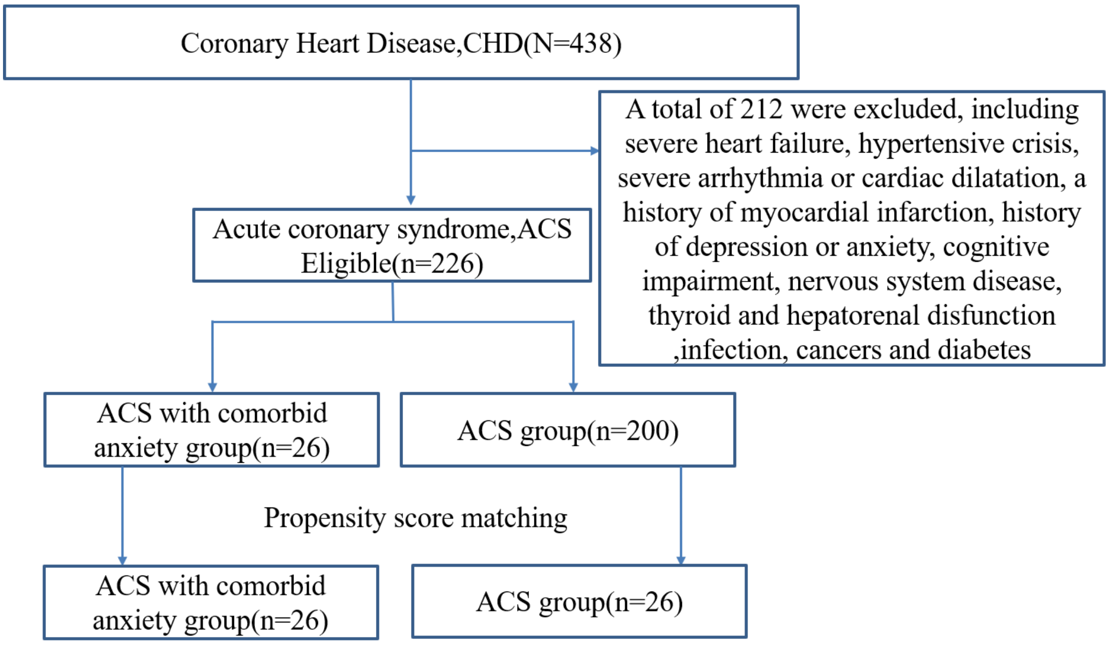

Supplementary Figure 2 The process of patient selection.
